# Robotic Total Pelvic Exenteration with Laparoscopic Rectus Flap: Initial Experience

**DOI:** 10.1155/2015/835425

**Published:** 2015-04-16

**Authors:** Brian R. Winters, Gary N. Mann, Otway Louie, Jonathan L. Wright

**Affiliations:** ^1^Department of Urology, University of Washington School of Medicine, Seattle, WA 98195, USA; ^2^Department of General Surgery, University of Washington School of Medicine, Seattle, WA 98195, USA; ^3^Department of Plastic Surgery, University of Washington School of Medicine, Seattle, WA 98195, USA; ^4^Division of Public Health Sciences, Fred Hutchinson Cancer Research Center, Seattle, WA 98109, USA

## Abstract

Total pelvic exenteration is a highly morbid procedure performed for locally advanced pelvic malignancies. We describe our experience with three patients who underwent robotic total pelvic exenteration with laparoscopic rectus flap and compare perioperative characteristics to our open experience. Demographic, tumor, operative, and perioperative factors were examined with descriptive statistics reported. Mean operative times were similar between the two groups. When compared to open total pelvic exenteration cases (*n* = 9), median estimated blood loss, ICU stay, and hospital stay were all decreased. These data show robotic pelvic exenteration with laparoscopic rectus flap is technically feasible. The surgery was well tolerated with low blood loss and comparable operative times to the open surgery. Further study is needed to confirm the oncologic efficacy and the suggested improvement in surgical morbidity.

## 1. Introduction

For patients with advanced primary and recurrent pelvic malignancies, total pelvic exenteration (TPE) involving en bloc resection of the rectum, bladder, and internal genital organs often provides the best chance of cure. Despite advancements in surgical techniques and perioperative care over time, TPE continues to be highly morbid with a wide range of complication rates (27–86%) [1–4]. The existing minimally invasive literature focuses primarily on anterior exenteration surgery while minimally invasive TPE (MITPE) data are lacking with no reports describing simultaneous laparoscopic rectus flap harvesting [5–9]. Herein, we report our initial experience with three cases of MITPE with laparoscopic rectus flap and compare perioperative characteristics to open TPE experience.

## 2. Case Reports

**Case 1 .**

57-year-old male with high-risk prostate cancer treated with high intensity focused ultrasound had early local recurrence with a large malignant rectourethral fistula. Fistula biopsy revealed recurrent prostate cancer extending to the rectal side of this fistula.

**Case 2 .**

78-year-old male with a history of prostate cancer was treated with brachytherapy presented 6 years later with cT4 high-grade, squamous differentiated urothelial carcinoma involving the bladder neck, prostate, and perirectal tissues.

**Case 3 .**

61-year-old male with T4N2M0 rectal adenocarcinoma treated with FOLFOXIRI therapy, followed by radiation therapy with adjuvant capecitabine. Further imaging revealed persistent mass involving the prostate, seminal vesicles, and bladder.

All MITPE patients were free of metastatic disease at time of surgery. MITPE surgeries were completed by a combined approach with Urology (Jonathan L. Wright), General Surgery (Gary N. Mann), and Plastic Surgery (Otway Louie). Open TPE cases performed (by Gary N. Mann) were identified over the preceding 6 years for comparison (8/2008–4/2014) (IRB#7968).

Robotic TPE was performed with the DaVinci SI system (Sunnyvale, CA; Intuitive Surgical, Inc.). Three robotic arms and two assistant ports were used in a configuration similar to robotic cystectomy with side docking to allow perineal access.

After initial mobilization of the sigmoid colon and ureteral ligation, the posterior dissection along the sacrum was taken distally and the lateral pedicles were then divided. The anterior attachments to the bladder were then taken down followed by ligation of the dorsal venous complex and urethral division. Circumferential mobilization of the rectum was continued distally until either a gastrointestinal anastomosis (GIA) stapler could be placed across the anorectal junction for en bloc resection (Cases 1 and 2) or a perineal incision could be made to complete the excision (Case 3).

Next, a laparoscopic, sheath-sparing, and rectus flap technique [10] was performed using the 3 existing left sided robotic ports as well as an additional LLQ 5 mm port. The peritoneum and posterior sheath were dissected off the rectus, preserving the deep inferior epigastric arteries, and the rectus was peeled off the anterior sheath with division of intercostal neurovascular bundles. The rectus was divided at the costal margin and placed in the pelvis for flap coverage.

The supraumbilical camera port was extended for removal of the specimen through an endoscopic surgical bag as appropriate. Ileal conduit and end colostomy were then performed through this minilaparotomy incision.

Baseline patient data included age, body mass index (BMI), Charlson score, albumin, and any previous abdominal surgery, chemotherapy, or radiation. Operative time and estimated blood loss (EBL) were recorded along with length of ICU stay, time to discharge, and short-term complications within one month of discharge. Duration of epidural/PCA usage as well as overall narcotic and analgesic usage was examined. Narcotic use was converted to IV morphine equivalents and summed for ease of comparison. Descriptive statistics are reported. Mean (standard deviation, SD) and median values between open and robotic cases are compared with *t*-tests and the nonparametric Wilcoxon rank-sum test, respectively. Categorical variables are compared with Chi square testing.

## 3. Discussion

Three MITPE cases are reviewed and compared to nine open TPE cases. Baseline characteristics are detailed in Table 1. There were no significant differences between combined MITPE and open TPE groups for any of the variables listed in Table 1 (*p* values all >0.20).

Operative times for MITPE cases were similar (9.5–11 hours) and not significantly different than the median operative times for open TPE (11.5 hours, *p* = 0.18) (Table 2). Six of nine open TPE patients had rectus flaps while the remaining three had omental flaps. Estimated blood loss (EBL) in MITPE patients ranged from 350 to 800 cc with transfusions of 2 units and 1 unit in Cases 1 and 2, respectively. Median EBL in MITPE patients was significantly lower than open TPE patients (500 cc versus 2300 cc, *p* = 0.01). There was one positive surgical margin in the MITPE group, which was not significantly different than positive margin rate in open TPE patients (*p* = 0.51).

All patients were taken to the ICU initially with median stay longer in the open TPE group (1 versus 3 days, *p* = 0.01). MITPE patients were discharged on PODs 7-8, which was significantly shorter than the median length of stay for open TPE patients (7 versus 13 days, resp., *p* = 0.01).

**Figure 1:**
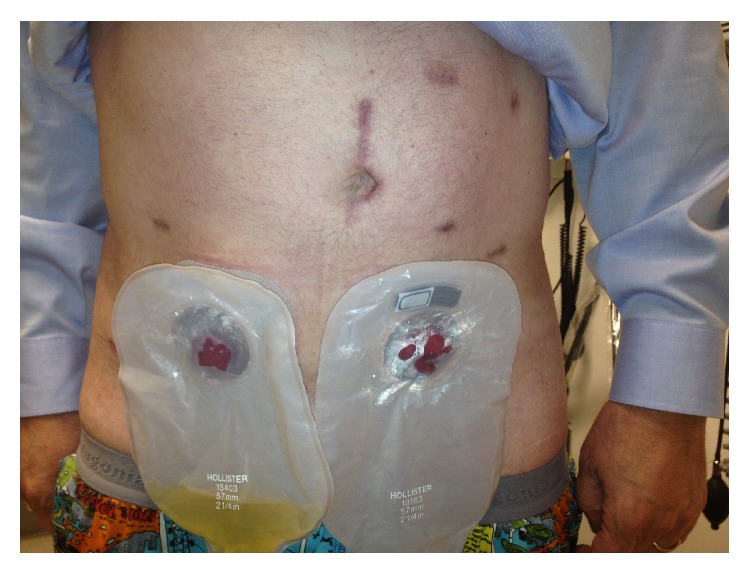
Postoperative surgical result: Case 2. Abdominal image of MITPE patient at one-month postoperative appointment revealing well-healed incisions with associated ileal conduit and end colostomy.

Table 2 reveals narcotic and analgesic usage between the two groups. Mean narcotic usage, measured in IV morphine equivalents, was less for MITPE than open TPE patients (99.8 (49) mg versus 961.1 (450) mg, resp., *p* = 0.09) with considerably more variance in the open TPE group. Other nonnarcotic analgesic usage was similar between groups.  Figure 1 shows MITPE patient (Case 2) at one-month postoperative visit.

Surgical complications were defined as any readmission within 1 month of discharge and were not significantly different between the two groups (*p* = 0.74). One MITPE patient was readmitted for pelvic abscess and pyelonephritis (Case 2). Open complications (44% of patients) included pelvic abscess (4), urosepsis (1), and* C. difficile* colitis (1). Longer-term complications in open TPE group included four patients with chronically draining perineal wounds and three patients with enterocutaneous fistulas. At time of analysis, follow-up of MITPE patients was insufficient to evaluate long-term complications; however, all three patients were back to daily activities within 4–6 weeks.

Overall, total pelvic exenteration remains a necessary component of oncologic surgery for treating pelvic malignancies. Despite advancements in surgical technique, the morbidity remains significant with complications ranging from 27 to 86% [1–4]. Data detailing robotic TPE surgery is limited and while the procedure has been described [7–9], to our knowledge, we are the first to describe perioperative characteristics of robotic versus open total pelvic exenteration. Further, we are the first to describe combining minimally invasive rectus flap coverage in this setting [10]. Our data suggest MITPE patients may experience similar operative times with lower blood loss, shorter ICU stays, less narcotic usage, and shorter hospital stays, although larger series are needed for more robust comparisons. In this analysis, the open TPE group had more adverse features (chemoradiation, higher Charlson scores, and BMI), limiting some ability to directly compare to the MITPE cases. Therefore, although these initial cases of MITPE demonstrate technical feasibility, selection bias may be present. Other limitations include small sample size, relatively short follow-up, and the retrospective nature of the analysis.

In conclusion, in well-selected patients, robotic TPE with laparoscopic rectus flap is a technically feasible surgical option that is well tolerated with low blood loss and short hospitalizations. Initial retrospective data appears promising but whether MITPE is as oncologically effective as open techniques will require increased surgeon experience and further study with long-term analysis.

## Figures and Tables

**Table 1:** Baseline characteristics of robotic total pelvic exenteration with laparoscopic rectus flap (MITPE) versus open patients.

	MITPE Case 1	MITPE Case 2	MITPE Case 3	Open TPE (9) median (range)
Age (years)	57	78	61	64 (47–75)
Previous abdominal surgery	Yes (colostomy)	No	Yes (colostomy)	6 (66.7%)
Previous radiation	No	Yes (brachytherapy)	Yes (external beam)	7 (77.8%)
Neoadjuvant chemotherapy	No	No	Yes	7 (77.8%)
Body mass index (BMI, kg/m^2^)	26.8	21.8	24	29.6 (20.6–40.3)
Charlson index	2	2	3	3 (2–6)
Preoperative albumin^†^ (normal: 3.4–5.4 g/dL)	Unavailable	2.9	1 (2 months before surgery)	3.1 (2.7–3.8)

Median values shown with percentages or ranges in parentheses as appropriate. MITPE: robotic total pelvic exenteration with laparoscopic rectus flap; TPE: total pelvic exenteration; kg/m^2^: kilograms/meter squared; g/dL: grams/deciliter.

^†^Preoperative albumin levels were available for 6 of 9 open TPE cases.

**Table 2:** Operative characteristics and hospital course of robotic total pelvic exenteration with laparoscopic rectus flap (MITPE) versus open patients.

	MITPE Case 1	MITPE Case 2	MITPE Case 3	Open TPE (9) median (range)
Operative time (hours)	11	10	9.5	11.5^†^ (8–14)
Rectus flap? (Yes/No)	Y	Y	Y	6 (66.7%)
Estimated blood loss (cc)^*^	800	500	350	2300 (950–6100)

	Infused narcotics (mg)			

Epidural use	None	None	5	4.5 (2–8)
PCA use	5	1	25.2	5^‡^ (1–14)

	IV morphine equivalents (mg)			

Overall narcotic usage	176.2	8.3	114.8	232.7 (34.8–3368.3)
	Mean = 99.8 (85)			Mean = 961.1 (1350)

	Other analgesic usage (mg)			

Ketorolac	240	None	None	75 (1 pt.)
Tylenol	650	16,900	14,000	1950 (650–16,250)
Others	None	None	Celecoxib 400; gabapentin 300	Ibuprofen 3600 (1800–3600, 3 pts^§^)

	Disposition			

ICU stay (POD)^*^	1	1	1	3 (2–14)
Discharge (POD)^*^	7	8	7	13 (8–17)

Median values for each parameter are shown with range or percentages in parentheses, unless otherwise labeled. Labeled means are shown with standard deviation (SD). Medication use pertains to in-hospital stay only. Narcotic use includes hydromorphone, oxycodone, oxycontin, and morphine converted to morphine equivalents and summed for comparison. MITPE: robotic total pelvic exenteration with laparoscopic rectus flap; TPE: total pelvic exenteration; POD: postoperative day; PCA: patient controlled analgesia; ICU: intensive care unit; cc: cubic centimeters.

^*^Statistically significant difference between MITPE and open TPE groups (*p* < 0.05).

^†^OR times available for 8 of 9 open TPE patients.

^‡^Two open exenteration patients were managed postoperatively with PCEA (patient controlled epidural analgesia) and therefore separate PCA use and total narcotic use relative to this was unavailable.

^§^Pts: number of patients in cohort who used this during hospital stay.
